# Multi-Level Image Thresholding Based on Modified Spherical Search Optimizer and Fuzzy Entropy

**DOI:** 10.3390/e22030328

**Published:** 2020-03-12

**Authors:** Husein S Naji Alwerfali, Mohammed A. A. Al-qaness, Mohamed Abd Elaziz, Ahmed A. Ewees, Diego Oliva, Songfeng Lu

**Affiliations:** 1 School of Computer Science and Technology, Huazhong University of Science and Technology, Wuhan 430074, China; i201722001@hust.edu.cn; 2 State Key Laboratory for Information Engineering in Surveying, Mapping and Remote Sensing, Wuhan University, Wuhan 430079, China; alqaness@whu.edu.cn; 3 Department of Mathematics, Faculty of Science, Zagazig University, Zagazig 44519, Egypt; abd_el_aziz_m@yahoo.com; 4 Department of Computer, Damietta University, Damietta 34517, Egypt; ewees@du.edu.eg; 5 Depto. de Ciencias Computacionales, Universidad de Guadalajara, CUCEI, Av. Revolución 1500, Guadalajara C.P. 44100, Jalisco, Mexico; diego.oliva@cucei.udg.mx; 6 Hubei Engineering Research Center on Big Data Security, School of Cyber Science and Engineering, Huazhong University of Science and Technology, Wuhan 430074, China; 7 Shenzhen Huazhong University of Science and Technology Research Institute, Shenzhen 518063, China

**Keywords:** image segmentation, multi-level thresholding, spherical search optimizer (SSO), sine cosine algorithm (SCA), fuzzy entropy, metaheuristics

## Abstract

Multi-level thresholding is one of the effective segmentation methods that have been applied in many applications. Traditional methods face challenges in determining the suitable threshold values; therefore, metaheuristic (MH) methods have been adopted to solve these challenges. In general, MH methods had been proposed by simulating natural behaviors of swarm ecosystems, such as birds, animals, and others. The current study proposes an alternative multi-level thresholding method based on a new MH method, a modified spherical search optimizer (SSO). This was performed by using the operators of the sine cosine algorithm (SCA) to enhance the exploitation ability of the SSO. Moreover, Fuzzy entropy is applied as the main fitness function to evaluate the quality of each solution inside the population of the proposed SSOSCA since Fuzzy entropy has established its performance in literature. Several images from the well-known Berkeley dataset were used to test and evaluate the proposed method. The evaluation outcomes approved that SSOSCA showed better performance than several existing methods according to different image segmentation measures.

## 1. Introduction

Image segmentation is a critical process in image processing technology which has been applied in various fields and applications, for example, in remote sensing [1], medical image [2], and others [3,4]. Image segmentation splits a given image into several classes that have similar properties, including color, brightness, gray level, contrast, and texture. There are different types of image segmentation techniques, including region extraction [5], clustering algorithms [6], edge detection [7], and thresholding [8]. Thresholding is an efficient segmentation method that asserts its efficiency in many applications. It is of two kinds, called bi-level (BLT) and multi-level thresholding (MLT). The BLT divides the objects of an image into two classes; therefore, if a given image has more classes, bi-level thresholding is not appropriate. MLT can solve this problem because it can divide the tested image into more classes. Previously, many multi-level thresholding methods had been applied using image histograms to get the best threshold values by maximizing or minimizing fitness functions (i.e., Otsu, and entropy).

However, traditional models face some limitations, such as computational time. Recently, metaheuristic (MH) methods have been widely applied to solve various optimization problems, including image segmentation. For example, Qi [8] presented a multi-level thresholding method based on particle swarm optimization (PSO) and maximum entropy. Different images, including remote sensing images, were utilized to test the improved PSO performance. In Reference [9], the authors proposed a segmentation method using multi-level thresholding. The galaxy-based search algorithm (GbSA) is applied to search for the optimal thresholding value, which is determined by maximizing Otsu’s criterion. The GbSA showed good performance in determining the optimal thresholding value. Mostafa et al. [10] used the whale optimization algorithm (WOA) to segment MRI images. WOA had been evaluated with various MRI images and asserts its efficiency in segmentation accuracy. In Reference [11], the authors presented a multi-level thresholding method based on moth-flame optimization (MFO). Both Otsu’s and Kapur’s entropy were used as the fitness function to evaluate the proposed method. Compared to PSO and bacterial foraging optimization (BFO), the MFO showed better performance. Social group optimization (SGO) [12] was applied for skin melanoma image segmentation. The firefly algorithm (FA) was applied for multi-level thresholding in Reference [13]. It applied Otsu as the objective function; also, the evolution results showed that FA had better performance compared to several existing methods. The FA also had been adopted in several multi-level thresholding [14,15,16]. Moreover, in Reference [17], both MFO and WOA were applied for multi-level thresholding. The evaluation experiments showed that MFO outperformed WOA. In Reference [18], the cuckoo search (CS) was applied for multi-level thresholding for gray-scale images. Also, in Reference [19], CS was applied for color images multi-level thresholding. Satapathy et al. [20] presented a multi-level thresholding approach based on the chaotic bat algorithm (CBA) and Otsu as a fitness function. CBA showed good performance compared to several methods. Also, Ant colony optimizer (ACO) was used for document image segmentation [21].

However, individual MH algorithms may be stuck at the local optima or may show slow convergence because some MH algorithms show good exploitation ability and some of them show good exploration ability [22]. To overcome these limitations, several hybrid metaheuristics have been proposed. For example, in Reference [23], a multi-level threshold method based on a hybrid of social spider optimization (SSO) and FA is presented. The developed FASSO method uses the power of both FA and SSO to avoid individual MH limitations. Mudhsh et al. [24] presented a hybrid of artificial bee colony (ABC) and FA to select the optimal threshold value by maximizing the Otsu function. This method was applied to enhance document image binarization and showed good performance. A hybrid approach of PSO and bacterial foraging optimization (BFO) for multi-level segmentation is presented in Reference [25]. This approach had been evaluated with eight images and reached good segmentation accuracy for both multi-level and bi-level thresholding. Another hybrid approach for multi-level thresholding is proposed by Reference [26] using the entropy function. The hybrid method is based on the gravitational search algorithm and genetic algorithm. In Reference [27], a hybrid multi-level thresholding method is proposed based on an improved salp swarm optimizer and Fuzzy entropy. The MFO is used to overcome the limitation of the salp swarm algorithm.

However, in such hybrid methods, one MH algorithm is needed to improve the local search for the other MH algorithm, such as, in Reference [27], the MFO is used as a local search for SSA. These hybrid MH methods can solve optimization problems efficiently. In the same context, we improved a new MH algorithm, called spherical search optimizer (SSO) [28], using the sine cosine algorithm (SCA), and applied the modified version, called SSOSCA, as an MLT image segmentation technique. In general, the SSO is based on the spherical search style, which is in contrast to the basic search style of previous MH algorithms. The SSO uses a combination of search styles to avoid the limitation of previous MH algorithms [28]. However, the exploitation ability of the SSO is less than its exploration ability. Therefore, we use the SCA to enhance it, since the SCA has the ability to exploit the search space and it has established its performance in different fields. The SCA is an efficient MH algorithm proposed by Reference [29]. In recent years, SCA has been applied in various optimization problems, such as in Reference [30], the SCA is employed to improve the adaptive neuro-fuzzy system (ANFIS) to forecast oil consumption in several countries. The SCA is employed to optimize the parameters of the ANFIS. In Reference [31], the authors applied SCA to enhance simulated annealing (SA) algorithm to build an efficient model for scheduling jobs in unrelated parallel machines that can be employed in manufacturing scheduling applications. In Reference [32], the SCA is applied to enhance the artificial bee colony (ABC) that applied for image segmentation. It is used to update individual solutions to find the optimal solution. In Reference [33], an improved SCA is proposed to solve global optimization problems. The improved SCA was evaluated using two popular benchmarks (CEC 2014 and CEC 2017) for various engineering problems, and it showed good performance. Also, in Reference [34], an improved SCA is proposed for solving global optimization problems. The opposition-based learning (OBL) is considered as a mechanism that improves the exploration of the search space to generate accurate solutions. A hybrid of SCA and genetic algorithm (GA) was proposed by Reference [35] for feature selection. Eight UCI datasets were used to evaluate the hybrid SCA and showed good performance. In Reference [36], a hybrid of atom search optimization and SCA is proposed for automatic data clustering. The SCA is employed as a local search method to enhance the performance of the atom search optimization.

In general, the proposed SSOSCA starts by setting the initial value for a set of agents depending on the computed histogram of the image of interest. Then, the Fuzzy entropy is employed to compute the quality of each agent since the Fuzzy entropy has a set of variant characteristics that made it suitable for the image segmentation problem. The next step is to search the agent which has the best fitness value, followed by updating the agents using the operators of SSO or SCA according to the probability of each solution that was computed depending on the fitness value of each agent. The process of searching for a suitable threshold is performed until the stopping conditions are met, and the best agent is considered as the output of the proposed SSOSCA.

Our main contributions are listed as follows:

1. We present an alternative multilevel thresholding technique based on modified MH algorithm, called spherical search optimizer (SSO). To the best of the authors’ knowledge, this is the first study that adopted SSO for image processing.
2. We enhance the exploitation ability of the SSO using the SCA’s operators.
3. We evaluate the performance of the SSOSCA using different images.
4. We compare the proposed SSOSCA with several existing methods.

The organization of this study is as follows: Section 2 presents the preliminaries of the problem definition, SSO and SCA. Section 3 presents a description of the proposed method. The evaluation and comparison experiment are presented in Section 4. We conclude this paper in Section 5.

## 2. Methodology

### 2.1. Problem Definition

The problem formulation of MLT is presented in this section. Assume we have a gray-scale image *I* which has K+1 classes. To divide a given image *I* into classes, the values of *k* thresholds $\left\{t_{k},k=1,2,K\right\}$ are needed, which can be defined as follows:

(1) $$\begin{array}{c} C_{0}\;=\left\{I_{ij}|0\leq I_{ij}\leq t_{1}-1\right\},\\ C_{1}\;=\left\{I_{ij}|t_{1}\leq I_{ij}\leq t_{2}-1\right\},\\ \ldots \\ C_{K}=\left\{I_{ij}|t_{K}\leq I_{ij}\leq L-1\right\} \end{array}$$

where *L* represents the maximum gray levels, $C_{K}$ is the *k*th class of the image, $t_{k}$ is the *k*th threshold, and $I_{ij}$ represents gray levels at the (i,j)th pixel and where the problem of the MLT can be defined as a maximization problem which is applied to find an optimal threshold value as follows:

(2) $$t_{1}^{*},t_{2}^{*},\ldots ,t_{K}^{*}=\arg\max_{t_{1},\ldots ,t_{K}}Fit\left(t_{1},\ldots ,t_{K}\right)$$

where Fit is the objective function. Here, the Fuzzy entropy [37] is applied as an objective function. Fuzzy entropy is a popular technology [38,39,40], which has been applied in many multi-level threshold segmentation applications, such as color images [41], brain tumor images [42], MRI images [43], and others [44,45]. It can be defined as follows:

(3) $$Fit\left(t_{1},\ldots ,t_{K}\right)=\sum_{k=1}^{K}H_{i}$$

(4) $$H_{k}=-\sum_{i=0}^{L-1}\frac{p_{i}\times \mu_{k}\left(i\right)}{P_{k}}\times ln\left(\frac{p_{i}\times \mu_{k}\left(i\right)}{P_{k}}\right),$$

(5) $$P_{k}=\sum_{i=0}^{L-1}p_{i}\times \mu_{k}\left(i\right)$$

(6) $$\mu_{1}\left(l\right)=\left\{\begin{array}{cc} 1 & l\leq a_{1}\\ \frac{l-c_{1}}{a_{1}-c_{1}} & a_{1}\leq l\leq c_{1}\\ 0 & l>c_{1} \end{array}\right.$$

(7) $$\mu_{K}\left(l\right)=\left\{\begin{array}{cc} 1 & l\leq a_{K-1}\\ \frac{l-a_{K}}{c_{K}-a_{K}} & a_{K-1}<l\leq c_{K-1}\\ 0 & l>c_{K-1} \end{array}\right.$$

$a_{1},c_{1},\ldots .,a_{k-1},c_{k-1}$ are the Fuzzy parameters, where $0\leq a_{1}\leq c_{1}\leq \ldots \leq a_{K-1}\leq c_{K-1}$. Then $t_{1}=\frac{a_{1}+c_{1}}{2},t_{2}=\frac{a_{2}+c_{2}}{2},\ldots ,t_{K-1}=\frac{a_{K-1}+c_{K-1}}{2}$.

### 2.2. Spherical Search Optimizer

In this section, the primary operators of the spherical search optimizer (SSO) are defined [28]. Two solutions *X* and *Y* are selected from the population *X* by the tournament selection method. Then, spherical search operators are used to update *X* using the following equations:

(8) $$X_{i}^{new}=F\times \left|\right|X_{p}-Y_{p}{\left|\right|}_{2}\times cos\left(\theta \right)$$

(9) $$X_{j}^{new}=F\times \left|\right|X_{p}-Y_{p}{\left|\right|}_{2}\times cos\left(\theta \right)sin\left(\omega \right)$$

(10) $$X_{k}^{new}=F\times \left|\right|X_{p}-Y_{p}{\left|\right|}_{2}\times sin\left(\theta \right)cos\left(\omega \right)$$

(11) $$X_{i}^{new}=F\times \left|\right|X_{p}-Y_{p}{\left|\right|}_{2}\times sin\left(\theta \right)$$

where i,j, and *k* are random selected integers representing the dimensions. *p* represents a set of integers (i.e., p=i,j,k). ${\left||.|\right|}_{2}$ refers to the $l_{2}$ norm (i.e., Euclidean distance). F∈[0,1] represents a scaling factor, and θ∈[0,π] is the angle between *X* and *Z*-axis. ω∈[0,2π] represents the angle in x˘y plane.

### 2.3. Sine Cosine Algorithm

Mirjalili [29] proposed the SCA as a population-based MH algorithm which uses sine and cosine functions to search for optimal solutions. The SCA begins by producing a group of *N* solutions represented as $X_{i},i=1,\cdots ,N$ in the following expression:

(12) $$X_{i}=l_{i}+rand\times \left(u_{i}-l_{i}\right)$$

where $l_{i}$ and $u_{i}$ are the lower and upper boundarues of the search domain, respectively. Thereafter, SCA computes its fitness function to evaluate each solution $X_{i}\in X$. The SCA updates the solution using one of its two main functions (sine or cosine) depending on the $r_{1}\in \left[0,1\right]$ probability random variable, as follows:

(13) $$X_{i}^{t+1}=\left\{\begin{array}{c} X_{i}^{t}+r_{2}\times sin\left(r_{3}\right)\times \left|r_{4}X_{b}^{t}-X_{i}^{t}\right|,\;r_{1}>0.5\\ X_{i}^{t}+r_{2}\times cos\left(r_{3}\right)\times \left|r_{4}X_{b}^{t}-X_{i}^{t}\right|,\;Otherwise \end{array}\right.$$

In Equation (13), $X_{b}$ refers to the best solution, where $r_{l}\in \left[0,1\right],l=2,3,4$ refers to a random number.

The goal of $r_{2}$ is to find the optimal area for updating *X*, which may be in the region between $X_{i}$ and $X_{b}$ or outside. Moreover, it is applied to balance exploitation and exploration by enhancing its values as follows [29]:

(14) $$r_{2}=a-t\frac{a}{t_{max}}$$

where *a* is a constant value, *t* is the current iteration, and $t_{max}$ is the maximum number of iterations. Furthermore, the goal of $r_{3}$ is to detect if $X_{i}$ moves to best solution $X_{b}$ direction or outwardly, where the goal of $r_{4}$ is to provide $X_{b}$ with a random weight to stochastically assert ($r_{4}>1$) or to stochastically de-assert ($r_{4}<1$) the effect of desalination in defining the distance.

## 3. Proposed Image Segmentation Method

**Figure 1:**
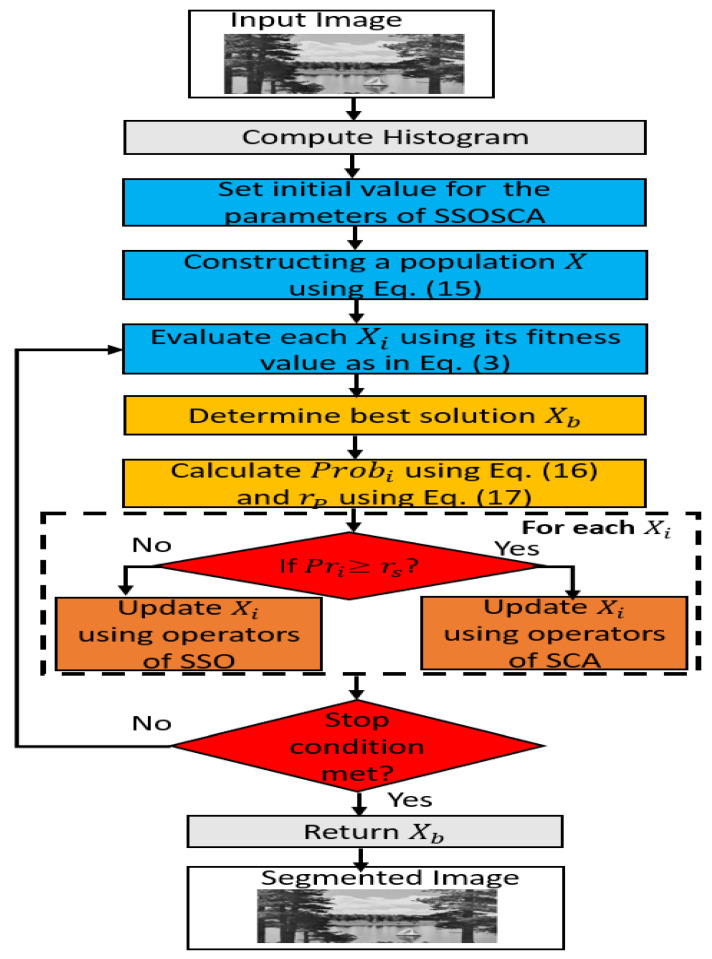
Steps of the SSOSCA method.

The steps of the proposed SSOSCA multilevel image segmentation technique are given in Figure 1. SSOSCA is an enhancement version of the traditional SSO algorithm based on the operators of SCA. This achieved by applying the SCA as a local search method for the SSO to improve its exploitation ability.

The steps of SSOSCA begin by setting the initial value randomly for a set of agents *X*, and this is performed using Equation (15).

(15) $$X_{i,j}=I_{min}+r_{1}\times \left(I_{max}-I_{min}\right),\;\;j=1,2,\ldots ,K,\;i=1,2,\ldots ,N$$

where $I_{max}$ and $I_{min}$ represent the largest and smallest gray values of the histogram of *I*, respectively. After that, SSOSCA assesses the quality of each agent based on its fitness value as defined in Equation (3), followed by allocating the best agent ($X_{b}$), which has a higher fitness value ($Fit_{b}$). The next step is to compute the probability $Pr_{i}$ for each agent depending on its fitness value $Fit_{i}$ as follows:

(16) $$Pr_{i}=\frac{Fit_{i}}{\sum_{i=1}^{N}Fit_{i}}$$

The operators of SSO are used to update the current agent $X_{i}$ (as defined in Equations (8)–(11)) in the case of $Pr_{i}<r_{s}$; otherwise, the two functions of SCA are used (i.e., sine and cosine) as defined in Equations (13) and (14). In this study, the value of $r_{s}$ is updated during the optimization process as follows:

(17) $$r_{s}=min\left(Pr_{i}\right)+rand\times \left(max\left(Pr_{i}\right)-min\left(Pr_{i}\right)\right),\;rand\in \left[0,1\right]$$

This strategy avoids the problem of determining the suitable value of $r_{s}$ to switch between the operators of SCA and SSO. The previous steps are performed again in case of the terminal conditions not being satisfied; otherwise, the best solution is returned, and this represents the best threshold value at a given threshold level. The quality of the segmented image is computed using suitable measures.

### Complexity of SSOSCA

The complexity of SSOSCA depends on the complexity of SSO and SCA. In general, the SSO has complexity $O(t_{max}\times N\times \left(4Dim\right))$ while the SCA has complexity $O(t_{max}\times N\times \left(Dim\right))$. Therefore, the complexity of SSOSCA is $O\left(t_{max}\times N_{SSO}\times \left(4Dim\right)\right)+O\left(t_{max}\times N_{SCA}\times \left(Dim\right)\right)$, where $N_{SSO}$ and $N_{SCA}$ are the number of solutions which updated using SSO and SCA, respectively.

## 4. Experiments and Results

**Figure 2:**
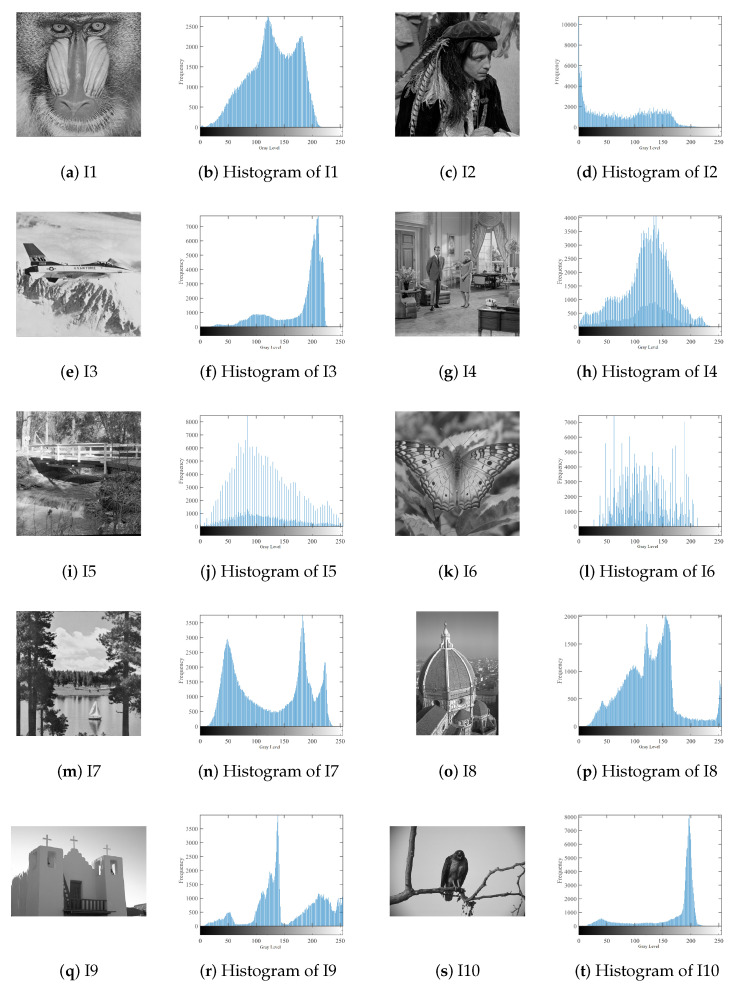
Histograms and original images.

To investigate the quality of the threshold obtained by the SSOSCA, ten images are used. These images have variant properties that can be observed from their histogram, as given in Figure 2.

### 4.1. Performance Measures

In order to assess the quality of the segmented image, a set of performance metric are used which includes Peak Signal-to-Noise Ratio (PSNR) [46,47] and the Structural Similarity Index (SSIM) [48]. PSNR and SSIM can be defined as follows:

(18) $$PSNR=20log_{10}\left(\frac{255}{RMSE}\right),\;RMSE=\sqrt{\frac{\sum_{i=1}^{N_{r}}\sum_{j=1}^{N_{c}}{\left(I_{i,j}-I_{S}i,j\right)}^{2}}{N_{r}\times N_{c}}}$$

where the RMSE is the root mean-squared error.

(19) $$SSIM\left(I,I_{S}\right)=\frac{\left(2\mu_{I}\mu_{I_{S}}+c_{1}\right)\left(2\sigma_{I,I_{S}}+c_{2}\right)}{\left(\mu_{I}^{2}+\mu_{I_{S}}^{2}+c_{1}\right)\left(\sigma_{I}^{2}+\sigma_{I_{S}}^{2}+c_{2}\right)}$$

$\mu_{I}$($\sigma_{I}$) and $\mu_{I_{S}}$ ($\sigma_{I_{S}}$) refer to the images’ mean intensity (standard deviation) of *I* and $I_{S}$, respectively. $\sigma_{I,I_{S}}$ is the covariance of *I* and $I_{S}$, and $c_{1}=6.5025$ and $c_{2}=58.52252$. Furthermore, we use the fitness value to evaluate the quality of threshold values; also, we use the CPU time for each algorithm.

### 4.2. Algorithms Comparison and Parameters Setting

In this section, the proposed SSOSCA is compared with other six approaches, including cuckoo search (CS) [49], grey wolf optimization (GWO) [50], whale optimization (WOA) [51], salp swarm algorithm (SSA) [52], grasshopper optimization algorithm (GOA) [53], and spherical search optimization (SSO). During fair comparisons, we set the size of the population and the number of the iterations to 20 and 100, respectively. The parameters of each approach are set to the original implementation of each approach. In addition, the parameters of SCA used in the proposed method are set according to the try and error method. However, we found that the parameters used on the original SCA references are more suitable and stable.

### 4.3. Results and Discussion

**Figure 3:**
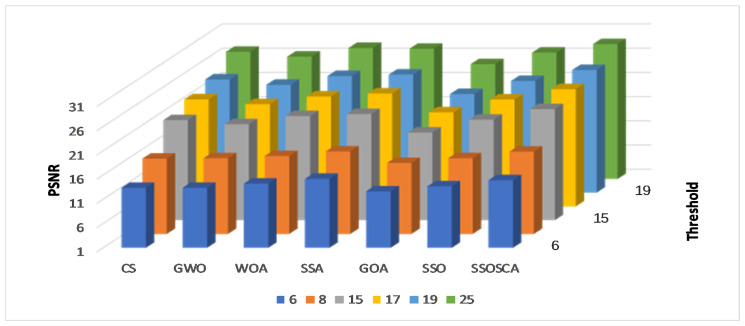
Results at each threshold in terms of Peak Signal-to-Noise Ratio (PSNR).

**Figure 4:**
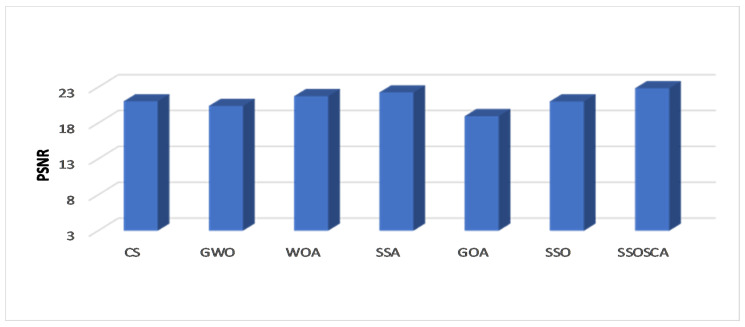
Average overall images in terms of PSNR.

**Figure 5:**
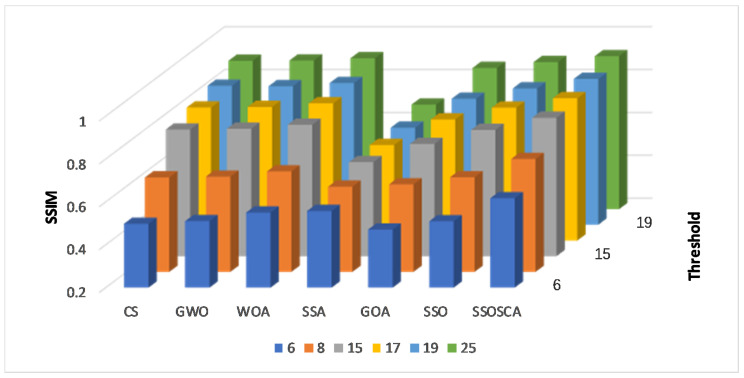
Results at each threshold level in terms of Structural Similarity Index (SSIM).

**Figure 6:**
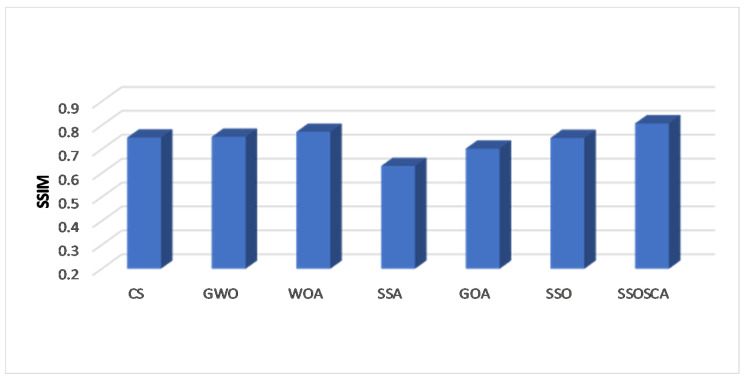
Average overall images in terms of SSIM.

We compare the SSOSCA approach to other approaches at different levels of the threshold, including 6, 8, 15, 17, 19, and 25. These values are considered higher with respect to other works, and they are used to assess the ability of the algorithms to determine the threshold values at these high levels. Since this is more suitable in real-world image processing applications, for example, remote sensing, medical images, and other cell images that have many objects. Table 1, Table 2 and Table 3 and Figure 3, Figure 4, Figure 5 and Figure 6 show the results of each approach at different threshold levels.

Table 1 illustrates the average of the PSNR at different threshold levels and among the ten tested images. From these results, we can see that the SSOSCA has a high ability to obtain the best threshold values that improve the segmentation of the given images. This is clear from the results where the SSOSCA has high PSNR values in forty-seven cases (as given in boldface) from the total sixty cases (ten image × six threshold levels). Followed by the SSA algorithm with eleven cases, while the WOA allocates the third rank with only two cases. Moreover, to study the performance of the algorithms at each threshold level, Figure 3 depicts the average of PSNR at each threshold level overall the ten images. From these average results, it can be noticed that SSA allocates the first rank at the two low threshold levels 6 and 8, followed by the proposed SSOSCA. Whereas at the higher threshold levels (i.e., 15, 17, 19, and 25) the proposed SSOSCA provides the best average, followed by SSA at levels 15, 17, and 19. At level 25, the WOA allocates the second rank. Moreover, Figure 4 shows the average of PSNR for each algorithm overall tested images and threshold levels, and we can see that the SSOSCA has the highest average of PSNR followed by SSA, while GOA, in this study, provides the worst PSNR value.

By analyzing the results of SSIM for each algorithm as given in Table 2, it can be observed that the segmented images using the obtained threshold values from the proposed SSOSCA are most similar to the original images. Therefore, the proposed SSOSCA has the highest SSIM at nearly forty-nine cases followed by WOA, SSA, and SSO in second, third, and fourth, respectively, with eight, two, and one case. The average of each algorithm at each threshold level is represented in Figure 5, and it can be seen that the SSOSCA provides the best average at all the tested threshold values. The WOA is the second best according to average of SSIM. Moreover, Figure 6 depicts the average overall the tested threshold and images; from this figure, we can conclude that the higher average of SSIM is achieved by the proposed SSOSCA followed by the WOA.

**Figure 7:**
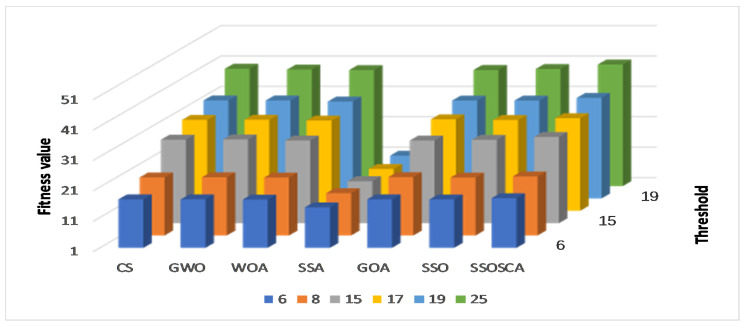
Results of at each threshold in terms of fitness value.

**Figure 8:**
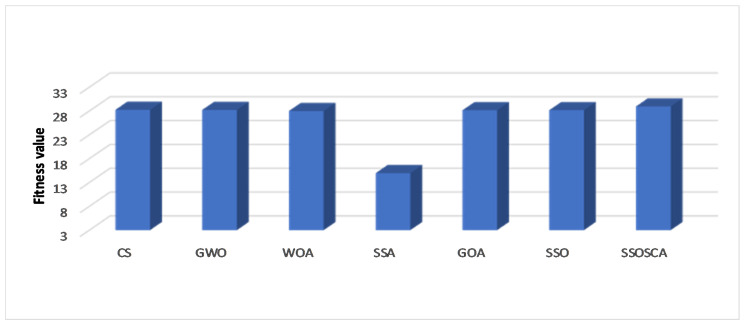
Average overall images in terms of fitness value.

According to the fitness value obtained by each algorithm, as shown in Table 3, it can be seen that the proposed SSOSCA has higher Fuzzy entropy value in thirty-three cases, nearly 55% from the total cases. Where the GOA allocates the second rank in terms of the fitness value with twenty-seven cases (45% from the total cases). From Figure 7, we can notice the high performance of the SSOSCA reaches the high fitness value at each tested threshold level, while, from Figure 8, it can be seen that most of the algorithms are competitive according to the average of the fitness values overall tested images and threshold values; however, the proposed SSOSCA takes the first rank with nearly 28.94, followed by CS with 28.235.

To analyze the performance of the CPU time(s) according to the CPU time(s) as given in Table 4, one can observe that the proposed SSOSCA has the smallest CPU time(s) in twenty-one cases from the sixty cases, followed by GWO and WOA, which take the second and third ranks, while the SSO that achieves the fourth rank.

**Figure 9:**
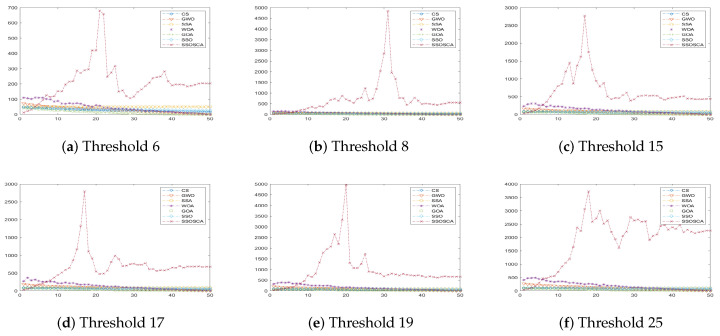
Diversity of the algorithms for image I1 at the tested threshold levels.

Figure 9 depicts the diversity of the proposed SSOSCA image segmentation approach at tested threshold levels for image I1. From this figure, it can be noticed that the SSOSCA maintains its diversity during the optimization while the diversity of other methods decreases with increasing iterations.

**Figure 10:**
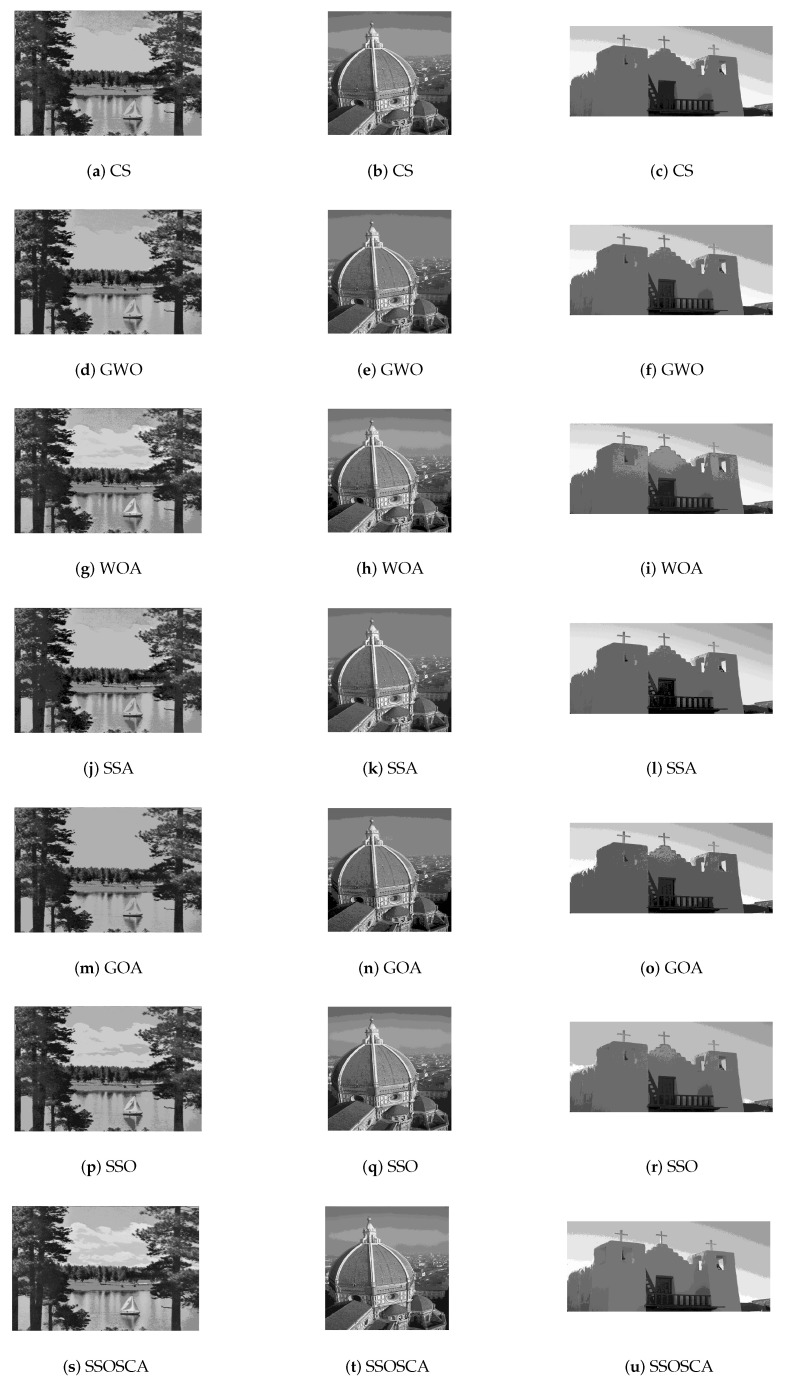
Segmented images at threshold value 19 for images I7–I9.

**Figure 11:**
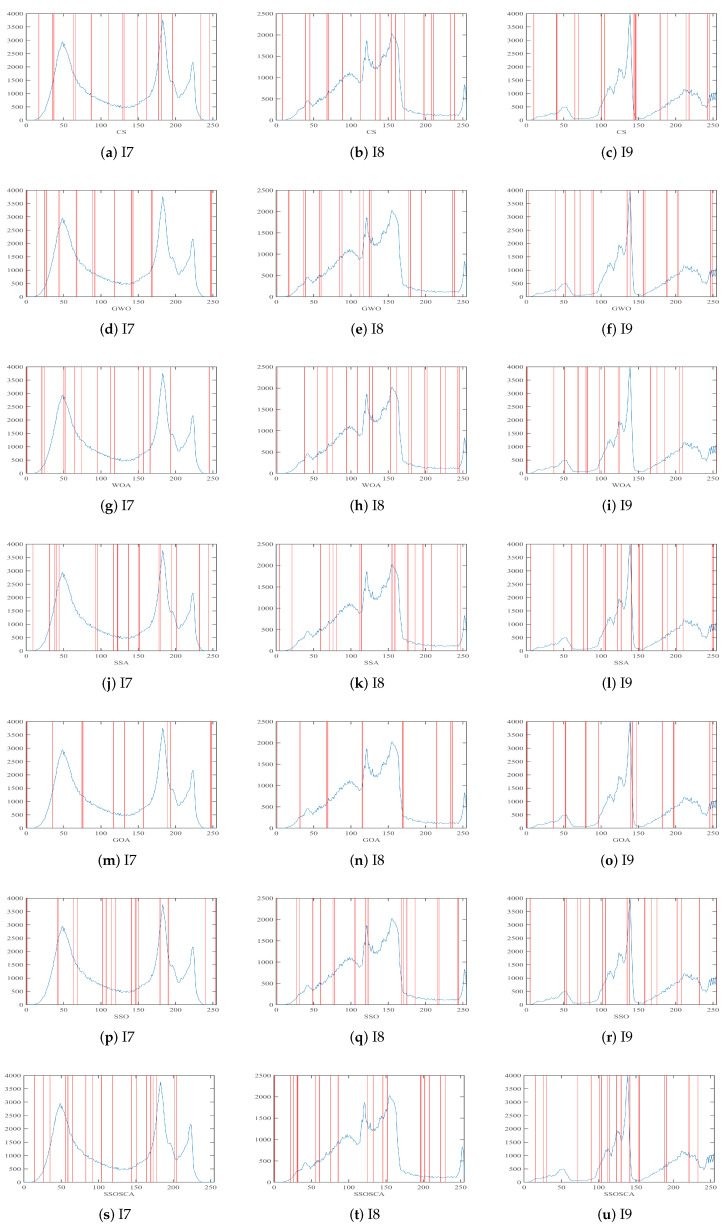
Threshold values obtained by each algorithm over the histogram of images I7–I9.

Figure 10 and Figure 11 show segmented images and their histogram of threshold values at threshold level 16. From the resulting images, we can see that, by using the threshold value obtained by the proposed SSOSCA, we can get high-quality segmented images.

### 4.4. Statistical Analysis using Friedman’s Test

In this section, we used the nonparameterize test, called Friedman test (FD), to study the robustness of the algorithms at the tested cases. The FD gives a statistical value that indicates the rank of the algorithm over all the tested algorithms, where a high rank refers to the best algorithm. The results of the obtained mean rank using FD is given in Table 5. It can be noticed that, in terms of PSNR, the SSOSCA has the highest mean rank among the three measures (i.e., fitness value, PSNR, and SSIM) while the SSA, CS, and GOA have the second best mean ranks in terms of PSNR, SSIM, and fitness value, respectively. Therefore, the threshold values obtained by SSOSSA is better than other algorithms, which enhances image segmentation.

To sum up, the comparison results showed that the SSOSCA has a high ability to find the threshold value that will lead to improving the quality of the segmented images. This can be observed from the values of fitness value, PSNR, and SSIM of the proposed SSOSCA. The main reason for this high quality is that the SSOSCA combines the operators of SSO to exploration the search space as well as the operators of SCA, which have high exploitation ability. This can be shown from the diversity of the proposed SSOSCA. However, the proposed SSOSCA still needs some improvements since its computational time is larger than some other methods since the original SSO has high complexity.

## 5. Conclusions

This study proposes an alternative multi-level thresholding segmentation method using a new metaheuristic called spherical search optimizer (SSO) and Fuzzy entropy. The proposed method, called SSOSCA, depends on a modified SSO algorithm using the sine cosine algorithm (SCA). To test the performance of the SSO method, we implement two experiments. We used ten images from the Brekely benchmark. The evaluation outcomes assess the efficiency of the proposed SSOSCA for image segmentation. Moreover, we compared the proposed method to several metaheuristics, such as CS, GWO, WOA, SSA, GOA, and SSO. Overall, the results showed that the proposed SSO outperforms other methods in terms of fitness value, PSNR, and SSIM. Furthermore, by concluding the high performance of the SSO, in future work, it may be applied in several optimization problems, such as time series forecasting, cloud computing, feature selection, and others. However, the proposed SSOSCA has some shortcomings that result from the traditional SSO algorithm. For example, the CPU time(s) needs to be improved, and this can be improved by replacing some operators. Moreover, the diversity of the proposed SSOSCA at some images degrades when the algorithm approaches the end of the iterations. These limitations open new directions to improve the performance of the SSOSCA, and this can be achieved by using disrupt operators, which established its ability to balance between exploration and exploitation in search space. After fixing these shortcomings, the proposed model can be developed as a multi-objective method and can be applied to other applications.

## Figures and Tables

**Table 1:** PSNR value for each algorithm.

Threshold	Image	CS	GWO	WOA	SSA	GOA	SSO	SSOSCA
6	I1	14.25352	14.12297	14.88168	**16.01488**	13.56229	14.59849	15.35288
	I2	15.88149	15.6123	15.89529	17.46374	15.45477	15.96416	**17.77297**
	I3	12.88066	12.60541	**14.19501**	13.69511	11.33032	13.48346	12.0036
	I4	16.2105	16.33116	15.78365	15.97745	16.0247	16.00978	**16.54122**
	I5	11.66614	11.90346	13.19066	13.95902	10.88047	12.56342	**15.03747**
	I6	11.92436	12.18304	13.55097	14.59268	11.50657	12.6031	**15.59027**
	I7	11.98298	11.82166	12.42468	**14.40488**	11.68666	12.33368	14.12488
	I8	14.48941	14.01864	14.505	16.09438	13.39676	14.17169	**16.27027**
	I9	10.15137	10.59881	12.06923	13.1789	9.386318	10.1912	**13.56904**
	I10	14.21204	14.42384	15.83079	**16.71505**	13.07327	14.70213	13.16674
	I1	18.15116	17.7062	17.5918	18.70303	17.05116	18.16227	**19.61937**
8	I2	16.89435	16.53922	17.96233	19.31846	15.64257	17.0666	**20.6029**
	I3	15.71986	15.96362	16.47572	**17.82194**	14.90901	16.56062	14.58805
	I4	17.6977	17.06365	17.20601	18.29229	16.77651	17.73637	**19.28856**
	I5	16.01255	16.15725	16.13152	16.78673	15.74769	15.72343	**18.64564**
	I6	15.18368	15.58474	18.37308	17.5583	14.06642	15.74106	**18.67142**
	I7	15.99579	15.54389	16.327	17.19466	15.13876	16.23942	**17.56659**
	I8	15.15281	16.89891	15.8369	17.48643	14.70882	15.0617	**19.15575**
	I9	15.50424	15.42388	16.43235	**17.59215**	14.23721	15.66292	16.33489
	I10	19.10778	19.31603	18.55335	**19.48182**	18.18214	18.33839	15.40225
	I1	23.01316	21.50909	22.73873	23.22639	20.8354	22.86816	**24.97524**
15	I2	22.437	22.18701	23.0264	23.2635	20.03461	22.45718	**25.21844**
	I3	21.52816	19.66725	22.92297	**24.01053**	19.29856	21.92662	23.73693
	I4	21.66735	21.68472	21.90825	23.11787	19.88215	22.5474	**23.98321**
	I5	21.16473	21.29523	21.9837	22.38433	18.60897	21.14932	**22.93739**
	I6	21.15053	20.50991	24.06414	23.81693	17.75073	21.95067	**24.95809**
	I7	21.32372	20.22902	20.9587	21.88487	18.4218	21.54657	**22.95848**
	I8	21.82251	21.29881	22.92123	22.66514	18.72172	21.60129	**24.01843**
	I9	20.96946	18.09634	20.8369	22.03847	17.77476	19.94986	**23.02196**
	I10	21.45881	21.4665	23.31075	22.62927	19.49201	21.41612	**23.42886**
	I1	24.52948	23.07464	23.75871	24.65909	22.31508	24.23343	**25.13423**
17	I2	24.1457	24.04834	24.31117	25.09358	20.85469	23.83807	**26.31966**
	I3	23.32701	20.65772	23.63131	**24.59668**	20.90329	23.35633	24.11021
	I4	22.89425	22.4866	23.87289	24.09969	20.98464	22.88265	**25.7949**
	I5	22.68493	22.86791	23.9018	23.85361	20.36532	22.29915	**25.20473**
	I6	22.21259	22.15515	24.99201	25.30651	19.23068	23.94462	**25.48218**
	I7	22.61354	21.41383	23.11234	**24.06132**	20.14508	22.1639	24.02152
	I8	22.68091	22.88689	22.79818	23.96688	19.94347	23.2374	**25.50871**
	I9	22.70389	19.35604	21.51394	23.23482	18.91577	21.63497	**24.52887**
	I10	23.15474	21.93014	24.80971	24.07668	20.54155	23.02558	**25.79065**
	I1	25.2359	24.25102	25.54807	26.02841	23.0769	25.15132	**26.94933**
19	I2	25.34997	24.97083	25.29321	26.31069	22.27348	24.5691	**27.99376**
	I3	24.74301	21.78626	24.6957	**25.62842**	21.58324	25.12392	**24.97041**
	I4	23.70948	23.91325	24.82184	24.29209	21.43777	23.34199	26.71273
	I5	24.15407	23.85685	24.83173	24.74708	21.7525	23.17846	**25.83682**
	I6	24.85081	23.62329	26.72128	26.55937	20.32694	24.04066	**26.80809**
	I7	24.53167	22.66648	23.76105	25.38036	21.27379	24.2728	**25.64757**
	I8	24.15169	23.87885	25.67028	24.90725	20.46545	24.15516	**26.19456**
	I9	22.5229	20.86362	23.67687	24.83083	19.78754	22.46845	**25.02367**
	I10	24.3168	22.75553	25.62639	24.85346	21.45175	24.12566	**26.93412**
	I1	27.40083	26.73225	28.64372	28.48424	25.75885	27.4094	**29.72896**
25	I2	28.22663	28.05796	28.57791	28.03914	26.21398	27.74724	**29.91905**
	I3	26.80269	23.93037	27.84109	**28.51179**	23.90788	27.44644	28.11514
	I4	26.75175	26.25728	27.00518	27.60792	24.95505	26.3363	**28.89368**
	I5	27.39457	26.90591	27.70828	27.55847	24.82644	26.32981	**28.65418**
	I6	26.74458	27.18045	**28.94175**	27.89996	23.77629	28.31972	28.8074
	I7	27.40609	25.97136	27.90512	27.36805	24.73083	26.79239	**28.66661**
	I8	27.20324	26.70906	27.15343	27.59151	24.63982	26.66865	**28.26119**
	I9	26.56469	24.43546	26.67952	26.70103	23.30677	25.72955	**27.32815**
	I10	27.66373	25.95623	29.52852	28.73986	24.66129	27.60036	**29.75223**

**Table 2:** SSIM values for each algorithm.

Threshold	Image	CS	GWO	WOA	SSA	GOA	SSO	SSOSCA
6	I1	0.523524	0.510253	0.568109	0.399377	0.489723	0.539125	**0.58022**
	I2	0.403976	0.402327	0.433124	**0.528733**	0.38488	0.408874	0.51094
	I3	0.61624	0.607159	0.643984	0.64755	0.612524	0.636622	**0.663075**
	I4	0.544795	0.551253	0.540463	0.525506	0.538583	0.535062	**0.57173**
	I5	0.299427	0.315309	0.401861	0.398346	0.246893	0.355741	**0.527396**
	I6	0.341501	0.361743	0.450753	0.390932	0.308731	0.384479	**0.538101**
	I7	0.419056	0.418795	0.450687	**0.545244**	0.395713	0.429664	0.540805
	I8	0.592113	0.572653	0.601576	0.645725	0.541014	0.579019	**0.675978**
	I9	0.577961	0.702382	0.718898	0.715349	0.564422	0.543722	**0.752861**
	I10	0.660338	0.661385	0.693721	0.779561	0.627041	0.683116	**0.806935**
	I1	0.714586	0.704407	0.700598	0.541409	0.680522	0.705873	**0.754957**
8	I2	0.45404	0.456672	0.531249	0.527609	0.403733	0.46437	**0.621204**
	I3	0.761106	0.752791	**0.77375**	0.658078	0.752033	0.776424	0.738899
	I4	0.600493	0.588868	0.59971	0.554734	0.573118	0.604819	**0.677128**
	I5	0.552234	0.565267	0.572991	0.52105	0.536587	0.533724	**0.695516**
	I6	0.511395	0.534196	0.654048	0.411087	0.446013	0.532338	**0.661291**
	I7	0.58497	0.568515	0.604636	0.587207	0.53671	0.589613	**0.678039**
	I8	0.647964	0.707133	0.66791	0.640183	0.636385	0.633611	**0.7744**
	I9	0.805569	0.804642	**0.818699**	0.743371	0.780551	0.805714	0.817822
	I10	0.777081	0.763212	0.765877	0.791986	0.738364	0.769774	**0.851697**
	I1	0.837799	0.812807	0.835306	0.604298	0.795047	0.835716	**0.881628**
15	I2	0.674244	0.703638	0.719994	0.588246	0.586459	0.664095	**0.762476**
	I3	0.854126	0.849842	0.868446	0.706102	0.826521	0.846497	**0.851063**
	I4	0.739509	0.748458	0.751812	0.584378	0.680759	0.760315	**0.806796**
	I5	0.762745	0.784467	0.796817	0.551934	0.672894	0.762572	**0.826962**
	I6	0.740847	0.72203	0.811659	0.433524	0.617372	0.755845	**0.825394**
	I7	0.767595	0.792932	0.762501	0.615011	0.646078	0.764162	**0.816658**
	I8	0.824795	0.835391	0.853562	0.68349	0.766416	0.825854	**0.878662**
	I9	0.848937	0.854201	0.860095	0.801097	0.824811	0.832077	**0.891477**
	I10	0.861982	0.846166	0.876463	0.819268	0.823452	0.845177	**0.91741**
	I1	0.871644	0.843402	0.858143	0.590451	0.830581	0.864172	**0.882139**
17	I2	0.734521	0.756154	0.745148	0.548721	0.615545	0.720611	**0.792555**
	I3	0.868686	0.866569	**0.875785**	0.720558	0.850289	0.87188	0.865206
	I4	0.773643	0.772249	0.800772	0.565424	0.72636	0.772247	**0.844171**
	I5	0.810143	0.827478	0.849047	0.606009	0.744826	0.801139	**0.876399**
	I6	0.777343	0.774738	0.838431	0.479682	0.676669	0.804816	**0.844831**
	I7	0.787594	0.819339	**0.823494**	0.622385	0.728787	0.781202	0.821508
	I8	0.841373	0.863496	0.852164	0.681778	0.792231	0.851523	**0.896992**
	I9	0.859167	0.854882	0.863581	0.811802	0.830793	0.855018	**0.896333**
	I10	0.884967	0.854984	0.89511	0.832219	0.853291	0.878345	**0.920322**
	I1	0.883	0.865282	0.889028	0.598864	0.847429	0.880617	**0.911516**
19	I2	0.764397	0.788061	0.772149	0.592437	0.668417	0.741729	**0.831151**
	I3	0.876207	0.874083	**0.897662**	0.699627	0.857745	0.879965	0.864455
	I4	0.792783	0.801929	0.824222	0.556423	0.734317	0.78558	**0.864702**
	I5	0.850908	0.852494	0.867394	0.576856	0.787629	0.824477	**0.891493**
	I6	0.836104	0.809292	0.863873	0.492018	0.714946	0.814909	**0.862724**
	I7	0.83388	0.840049	0.823023	0.646931	0.761484	0.82062	**0.862201**
	I8	0.876644	0.880924	0.891312	0.72755	0.806464	0.869286	**0.903824**
	I9	0.87026	0.871134	0.881606	0.811531	0.837176	0.869857	**0.899958**
	I10	0.905005	0.886993	0.904834	0.829729	0.870284	0.878774	**0.914785**
	I1	0.915084	0.905819	0.934535	0.641548	0.895104	0.91449	**0.942844**
25	I2	0.837243	0.864696	0.849402	0.637007	0.792308	0.819998	**0.875724**
	I3	0.904065	0.90137	**0.916284**	0.719599	0.883033	0.898039	0.897269
	I4	0.860557	0.854364	0.869306	0.608607	0.822635	0.851806	**0.900192**
	I5	0.912592	0.911123	0.918719	0.605743	0.866388	0.893672	**0.931443**
	I6	0.874735	0.886428	**0.904776**	0.576811	0.822266	0.891801	0.894003
	I7	0.879834	0.877669	0.897166	0.668648	0.843863	0.870174	**0.903471**
	I8	0.911805	0.914548	0.912151	0.75603	0.883773	0.903593	**0.935702**
	I9	0.903479	0.893403	0.905438	0.81819	0.874026	0.893238	**0.920015**
	I10	0.924179	0.922264	0.93366	0.843104	0.9025	0.926239	**0.934209**

**Table 3:** Fitness value for each algorithm.

Threshold	Image	CS	GWO	WOA	SSA	GOA	SSO	SSOSCA
6	I1	17.51627	17.52452	17.50563	14.55501	**17.53978**	17.45458	17.33092
	I2	17.29183	17.28936	17.24797	15.56939	**17.3161**	17.27256	17.28515
	I3	17.08744	17.08179	17.01854	13.96991	17.10156	17.06157	**17.31998**
	I4	17.55223	17.5704	17.5493	15.39157	**17.58961**	17.52835	17.28721
	I5	15.59818	15.59255	15.53775	12.72764	15.6182	15.63955	**17.3201**
	I6	15.07032	15.08058	15.02587	11.5213	15.12722	15.01716	**17.28756**
	I7	17.62055	17.62355	17.60271	15.13502	17.31648	17.4764	**17.64178**
	I8	17.57384	17.5903	17.50949	15.48919	**17.60093**	17.54151	17.26228
	I9	17.47719	17.50937	17.3717	14.95461	**17.53705**	17.47222	17.33736
	I10	16.76789	16.77492	16.68104	14.05739	16.79876	16.76765	**17.31798**
8	I1	20.77239	20.81951	20.79091	15.6432	**20.83927**	20.68942	20.36499
	I2	20.77715	20.81823	20.64625	16.20068	**20.914**	20.69229	20.40957
	I3	20.44303	20.4538	20.41782	14.46727	**20.5345**	20.38249	20.4722
	I4	20.91361	20.95094	20.88245	16.30784	**21.00918**	20.85257	20.45572
	I5	18.26216	18.32197	18.24171	14.13618	18.3769	18.26113	**20.38147**
	I6	17.38663	17.42614	17.20956	11.56811	17.50213	17.28073	**20.43823**
	I7	20.87007	20.91007	20.85901	15.3929	**20.94932**	20.82676	20.38342
	I8	20.87381	20.83527	20.83046	15.30596	**20.98815**	20.85887	20.41978
	I9	20.98318	21.03984	20.81272	15.67114	**21.05458**	20.98732	20.36542
	I10	19.97626	20.01734	19.86859	14.78115	20.06006	19.91779	**20.477**
15	I1	29.39068	29.46837	29.37888	16.21008	**29.80082**	29.27839	28.49456
	I2	29.68226	29.75557	29.54176	16.07059	28.55657	29.68748	**30.15125**
	I3	29.26056	29.26241	29.05608	14.08843	28.54654	29.13099	**29.78387**
	I4	29.53076	29.63429	29.35599	15.90209	**30.01778**	29.55382	28.53975
	I5	25.20403	25.2145	24.91564	13.76618	25.7182	25.2165	**28.49094**
	I6	23.63031	23.61669	22.53367	11.62479	24.23131	23.18178	**28.52712**
	I7	29.4742	29.59732	29.58911	14.90863	28.61348	29.41551	**30.03443**
	I8	30.06616	30.13922	29.64109	15.51657	28.64436	30.03488	**30.5571**
	I9	29.74802	30.00956	29.58872	15.29011	28.52038	29.90233	**30.4636**
	I10	28.86841	28.94542	28.53648	14.78096	**29.28097**	28.86354	28.53572
17	I1	31.95775	31.94438	31.99469	16.04241	31.07617	31.84159	**32.47225**
	I2	32.3915	32.43306	32.39763	15.71142	**33.00691**	32.41968	30.99624
	I3	31.78612	31.7911	31.61119	15.02817	**32.42836**	31.69459	31.00468
	I4	32.13392	32.13961	31.87661	16.45778	**32.75535**	32.17488	30.8959
	I5	27.16318	27.21435	26.99812	13.17875	27.73732	27.22226	**31.0181**
	I6	25.28239	25.28696	24.30813	11.52589	26.12225	24.6364	**30.95348**
	I7	32.10722	32.19439	32.10384	15.06927	**32.62854**	32.09904	31.07726
	I8	32.67831	32.70998	32.45816	15.26126	**33.33993**	32.65714	31.04546
	I9	32.44411	32.53263	32.15043	14.957	30.99357	32.46321	**33.17218**
	I10	31.46008	31.58081	31.07522	14.68681	31.0726	31.50329	**32.03533**
19	I1	34.36383	34.2342	34.21043	16.34169	33.28233	34.21749	**34.99164**
	I2	34.97506	34.97331	34.90153	16.38087	33.30956	35.0698	**35.71332**
	I3	34.21961	34.14	33.91049	14.96975	**35.06493**	34.09816	33.37397
	I4	34.66706	34.64812	34.41234	15.79635	**35.3929**	34.68254	33.23854
	I5	28.9961	29.03604	28.6912	13.67946	29.68399	29.14927	**33.31114**
	I6	26.75121	26.54048	25.45928	11.52925	27.53597	25.98213	**33.27359**
	I7	34.63642	34.73002	34.61115	15.36037	**35.32157**	34.55686	33.24501
	I8	35.20329	35.22804	34.99536	15.48853	**35.9805**	35.26575	33.35999
	I9	34.96186	35.02181	34.32166	15.99765	33.31905	35.06191	**35.77686**
	I10	33.9221	34.01549	33.57158	15.1945	33.31914	34.01515	**34.71253**
25	I1	41.07035	40.64346	40.86762	17.18634	39.56373	40.95834	**41.88894**
	I2	42.1871	41.86861	41.8537	16.67131	**42.9219**	42.13362	39.57032
	I3	40.60734	40.24792	40.15664	15.22677	**41.68104**	40.41704	39.63348
	I4	41.55509	41.22411	41.15408	17.3623	**42.46069**	41.68751	39.56037
	I5	33.83683	33.72248	33.42687	14.79988	34.74606	33.99238	**39.72255**
	I6	30.47008	29.62294	29.12014	12.21516	32.05033	29.29351	**39.7571**
	I7	41.59242	41.48565	41.40234	16.04463	39.54619	41.55445	**42.40039**
	I8	42.34127	42.11024	42.07119	16.37498	39.72544	42.35157	**43.03618**
	I9	41.8899	41.9941	41.43598	16.2916	39.55762	42.13391	**42.82789**
	I10	40.81655	40.49642	40.21611	15.92253	39.76649	40.77342	**41.79107**

**Table 4:** CPU time(s) for each algorithm.

		CS	GWO	WOA	SCA	GOA	SSO	SSOSCA
6	I1	0.5769	0.4637	**0.4547**	1.2575	0.4899	0.4677	0.4986
	I2	0.5830	0.4646	0.4650	1.2781	0.4976	0.4663	**0.4630**
	I3	0.5520	**0.4413**	0.4503	1.2531	0.4836	0.4508	0.4545
	I4	0.5442	0.4377	0.4361	1.2255	0.4583	0.4582	0.4672
	I5	0.5487	0.4484	0.4384	1.2456	0.4676	0.4514	**0.4359**
	I6	0.5610	**0.4517**	0.4590	1.2526	0.4760	0.4537	0.4697
	I7	0.5492	0.4414	**0.4403**	1.2423	0.4717	0.4496	0.4462
	I8	0.4562	**0.3472**	0.3480	1.1384	0.3697	0.3520	0.3556
	I9	0.4509	0.3398	**0.3369**	1.1356	0.3699	0.3456	0.3550
	I10	0.4950	0.3888	0.3923	1.2307	0.4109	0.3818	**0.3639**
8	I1	0.6297	0.4993	0.5033	1.4758	0.5261	**0.4984**	0.5273
	I2	0.6217	0.4843	**0.4804**	1.4581	0.5072	0.4881	0.5020
	I3	0.6104	0.4760	0.4756	1.4386	0.5060	0.4924	**0.4726**
	I4	0.5965	**0.4619**	0.4626	1.4034	0.4848	0.4825	0.4981
	I5	0.6224	0.4824	**0.4817**	1.4562	0.5081	0.4889	0.5264
	I6	0.6308	0.5039	0.4977	1.5069	0.5177	0.5024	**0.4816**
	I7	0.6162	0.4811	0.4815	1.4593	0.5103	0.4850	**0.4735**
	I8	0.5147	0.3862	**0.3840**	1.3623	0.4118	0.3866	0.3931
	I9	0.5427	0.3921	**0.3829**	1.3938	0.4190	0.3963	0.4011
	I10	0.5427	0.3926	**0.3816**	1.3639	0.4145	0.3929	0.4045
15	I1	0.8410	**0.5954**	0.6013	2.1359	0.6334	0.6105	0.6953
	I2	0.8407	0.5974	0.6035	2.1409	0.6253	0.5939	0.6062
	I3	0.8273	**0.5764**	0.5800	2.0536	0.6082	0.5898	0.5986
	I4	0.8236	0.5854	0.5931	2.0602	0.6066	0.5980	**0.5725**
	I5	0.8387	0.5844	0.5987	2.0817	0.6214	0.5934	**0.5796**
	I6	0.8608	0.6066	0.5993	2.1608	0.6387	0.6112	**0.5906**
	I7	0.8155	0.5842	0.5966	2.0720	0.6144	0.5966	**0.5824**
	I8	0.7050	0.4737	0.4785	1.9618	0.5040	0.4805	**0.4695**
	I9	0.7102	0.4692	0.4799	1.9408	0.5039	0.4755	0.5786
	I10	0.7054	0.4848	0.4752	1.9741	0.5205	0.4794	**0.4751**
17	I1	0.8932	**0.6258**	0.6424	2.2970	0.6659	0.6305	0.6867
	I2	0.8849	0.6256	0.6247	2.2696	0.6485	**0.6246**	0.6364
	I3	0.8801	**0.6176**	0.6221	2.2763	0.6354	0.6262	0.6345
	I4	0.9005	0.6386	0.6259	2.2988	0.6611	0.6344	**0.6155**
	I5	0.8855	0.6263	0.6200	2.3080	0.6494	0.6258	**0.6144**
	I6	0.8777	0.6186	0.6142	2.2895	0.6346	0.6333	**0.6114**
	I7	0.8972	**0.6245**	0.6272	2.3090	0.6566	0.6366	0.6593
	I8	0.7798	0.5273	0.5233	2.1741	0.5577	**0.5190**	0.5444
	I9	0.7724	0.5175	0.5151	2.1660	0.5490	0.5186	**0.5117**
	I10	0.7727	**0.5025**	0.5101	2.1519	0.5376	0.5081	0.5645
19	I1	0.9539	**0.6580**	0.6629	2.4952	0.6923	0.6638	0.6723
	I2	0.9572	0.6575	**0.6536**	2.5105	0.6873	0.6706	0.6640
	I3	0.9444	0.6430	0.6396	2.4728	0.6696	0.6571	**0.6268**
	I4	0.9495	**0.6456**	0.6495	2.4701	0.6757	0.6538	0.6691
	I5	0.9589	0.6650	**0.6623**	2.5054	0.6985	0.6663	0.6824
	I6	0.9650	0.6843	0.6793	2.5953	0.7130	**0.6748**	0.6860
	I7	0.9601	**0.6559**	0.6601	2.4579	0.6781	0.6586	0.7493
	I8	0.8373	0.5410	0.5469	2.3879	0.5734	0.5607	**0.5375**
	I9	0.9081	0.5836	0.6010	2.5097	0.6124	**0.5679**	0.6085
	I10	0.8162	**0.5307**	0.5421	2.3427	0.5647	0.5379	0.5942
25	I1	1.1320	**0.7448**	0.7553	3.1039	0.7806	0.7577	0.8425
	I2	1.1306	0.7578	**0.7513**	3.1007	0.7702	0.7643	0.7696
	I3	1.1387	0.7417	0.7554	3.1092	0.7717	0.7522	0.8364
	I4	1.1279	0.7467	**0.7393**	3.0678	0.7774	0.7442	0.7600
	I5	1.1876	0.7839	0.7739	3.1912	0.8088	**0.7713**	0.8026
	I6	1.1602	**0.7610**	0.7665	3.1010	0.7918	0.7794	0.8071
	I7	1.1364	0.8219	0.7543	3.0817	0.7810	0.7615	**0.7534**
	I8	1.0198	0.7015	0.6434	3.0088	0.6793	0.6451	**0.6404**
	I9	1.1658	0.7380	0.7069	3.2423	0.7622	0.7224	**0.6870**
	I10	1.0496	0.6523	**0.6466**	3.0418	0.6764	0.6624	0.7263

**Table 5:** Results of the Friedman test.

	CS	GWO	WOA	SSA	GOA	SSO	SSOSCA
PSNR	3.5833	2.7166	4.85	5.76666	1.11666	3.516	6.45
SSIM	4.2166	4.0166	5.6	1.9	1.88333	3.8	6.583
Fitness	4.65	5.05	3.0333	1	5.23333	4.183	4.85
CPU time(s)	6	2.1667	2.1083	7	4.7000	2.8417	3.1833
